# Reduced Numbers and Impaired Function of Regulatory T Cells in Peripheral Blood of Ischemic Stroke Patients

**DOI:** 10.1155/2016/2974605

**Published:** 2016-03-17

**Authors:** Johanna Ruhnau, Juliane Schulze, Bettina von Sarnowski, Marie Heinrich, Sönke Langner, Christian Pötschke, Anika Wilden, Christof Kessler, Barbara M. Bröker, Antje Vogelgesang, Alexander Dressel

**Affiliations:** ^1^Department of Neurology, University Medicine Greifswald, 17475 Greifswald, Germany; ^2^Department of Diagnostic Radiology and Neuroradiology, University Medicine Greifswald, 17475 Greifswald, Germany; ^3^Department of Immunology, University Medicine Greifswald, 17475 Greifswald, Germany

## Abstract

*Background and Purpose*. Regulatory T cells (Tregs) have been suggested to modulate stroke-induced immune responses. However, analyses of Tregs in patients and in experimental stroke have yielded contradictory findings. We performed the current study to assess the regulation and function of Tregs in peripheral blood of stroke patients. Age dependent expression of CD39 on Tregs was quantified in mice and men.* Methods*. Total FoxP3^+^ Tregs and CD39^+^FoxP3^+^ Tregs were quantified by flow cytometry in controls and stroke patients on admission and on days 1, 3, 5, and 7 thereafter. Treg function was assessed by quantifying the inhibition of activation-induced expression of CD69 and CD154 on T effector cells (Teffs).* Results*. Total Tregs accounted for 5.0% of CD4^+^ T cells in controls and <2.8% in stroke patients on admission. They remained below control values until day 7. CD39^+^ Tregs were most strongly reduced in stroke patients. On day 3 the Treg-mediated inhibition of CD154 upregulation on CD4^+^ Teff was impaired in stroke patients. CD39 expression on Treg increased with age in peripheral blood of mice and men.* Conclusion.* We demonstrate a loss of active FoxP3^+^CD39^+^ Tregs from stroke patient's peripheral blood. The suppressive Treg function of remaining Tregs is impaired after stroke.

## 1. Introduction 

Ischemic stroke-induced immune alterations (SIIA) are thought to affect the outcome of stroke patients, in part by enhancing their susceptibility to bacterial infection. This has been shown in experimental stroke models and in patients [1–3]. The alterations in the immune system are thought to be mediated by rapid activation of the autonomic nervous system and the hypothalamic-pituitary-adrenal (HPA) axis. In stroke patients, plasma levels of stress hormones correlate with the extent of immune alterations [4, 5].

In addition to the systemic immune-suppressive alterations observed in the peripheral blood, a local inflammatory immune response also develops. Within 24 h, leukocytes accumulate in the ischemic brain region [6, 7]. Recent observations suggest that lymphocytes but not granulocytes can trespass the blood brain barrier and infiltrate the ischemic lesion and the penumbra [8–10]. To date, the mechanisms that regulate this local inflammatory response and the role of the leukocyte subtypes involved are only partially understood. In experimental stroke models, T cells sensitized to CNS antigens have been transferred into mice lacking B and T cells. These autoreactive T cells were found in the stroke lesion, and their transfer enhanced the severity of stroke [11]. Furthermore, immunological tolerization of T cells to CNS autoantigens has beneficial effects on experimental stroke outcome [12]. Together, these data suggest that local inflammation following stroke has some autoimmune properties.

Regulatory T cells (Tregs) are modulators of adaptive immune responses and play an important role in maintaining tolerance to self-antigens. Depletion of murine CD25^+^CD4^+^ Tregs or abrogation of their function exacerbates various autoimmune diseases, including autoimmune gastritis, thyroiditis, and type 1 diabetes [13]. Thus, it has been hypothesized that, in stroke, Tregs may dampen the immunological cascades that result in secondary brain damage, which is not directly caused by ischemia. This concept is supported by the findings of Liesz et al., who used antibody-mediated depletion of CD25^+^ cells to eliminate Tregs and reported worse outcome in an experimental stroke model [14]. In contrast, Kleinschnitz et al. have reported that depleting FoxP3^+^ Tregs with an inducible diphtheria toxin receptor construct under the control of the FoxP3 promoter decreased brain damage [15]. Additional conflicting data have been reported, wherein Treg depletion using a similar mouse model in a different experimental stroke model did not alter the infarct volume within the initial 4 days after stroke [16]. More recently, controversial data continue to be reported from experimental stroke models. Boosting Treg function with a superagonistic anti-CD28 antibody (CD28SA) 3 h after middle cerebral artery occlusion (MCAO) reduced brain damage and improved outcome, while pretreatment with the same CD28SA antibody worsened clinical outcome and treatment immediately after MCAO had no effect on the ischemic brain volume in another study [17, 18]. In humans, Treg enumeration and function in peripheral blood have also yielded contradictory results. While Hug et al. were unable to detect changes in Treg function in patients with ischemic stroke [5], Yan et al. reported impaired function, but an increased percentage of Tregs [19].

In part, these contradictory findings could result from the fact that even FoxP3^+^ Tregs are not a homogenous population; instead, they can be divided into several subgroups that differ in function and can be distinguished by surface antigen expression [20]. CD45RA is expressed on naive FoxP3^+^ cells. Even in adulthood, some naive FoxP3^+^ cells can be found in the circulation. Upon antigen stimulation, CD45RA^+^ Tregs lose their CD45RA expression, start to proliferate, and differentiate to a more-suppressive Treg phenotype [13, 20]. CD39 is a rate-limiting ectonucleotidase that cleaves the proinflammatory extracellular adenosine triphosphate (ATP) [21, 22] to inhibitory and antiproliferative adenosine monophosphate [23]. Indeed, expression of CD39 has been shown to identify functionally active, suppressive Tregs in rodents and humans [24].

We performed the current study to determine the regulation of Treg subsets and Treg function in the peripheral blood of human stroke patients.

## 2. Methods

### 2.1. Human Studies

#### 2.1.1. Patients and Controls

Patients (age: >18 y) with acute middle cerebral artery infarction were eligible for the study within 12 h of disease onset. They were recruited at the Department of Neurology of the University Medicine Greifswald if their National Institutes of Health Stroke Scale (NIHSS) score was ≥6, they had no signs of infection on admission, and their plasma levels of C-reactive protein (CRP) were ≤50 mg/L and of procalcitonin (PCT) ≤0.5 ng/mL. Patients were excluded if they took immune-suppressive drugs, suffered from known malignancies, or had an NIHSS score of <6. Treatment complied with best medical care standards and took place in a dedicated stroke unit. Recombinant tissue plasminogen activator administration and thrombectomy took place as clinically indicated. Control subjects were either healthy or recruited from the Ophthalmology Clinics at the University Medicine Greifswald. Control subjects were of similar age and had no known neurological or immunological disorders and fulfilled the same criteria for CRP and PCT as stroke patients. Patient and control characteristics are listed in Table 1(a). In addition a cohort of 32 younger healthy controls (age 21–79 years) was recruited to analyze age dependency of CD39 expression on Treg.

Patients were allocated into the stroke associated infection (SAI+) cohort if they had developed (a) clinical signs of infection (pneumonia, urinary tract infections, and fever of unknown origin); (b) serum concentrations of CRP >50 mg/L; and (c) PCT serum concentrations >0.5 ng/mL. Only patients that matched none of the criteria throughout the whole study period were considered free of infection (SAI−). Patients who fulfilled some criteria but not all were excluded from the comparison of stroke patients with and without SAI. These criteria were designed to identify two distinct populations of patients as published previously [3]. Details on SAI+ and SAI− patients are given in Table 1(b).

#### 2.1.2. Ethics Statement

The study protocol was approved by the ethics committee of the Medical Faculty, University of Greifswald (No. III UV 30/01). All patients gave written informed consent directly or through a surrogate where appropriate.

#### 2.1.3. CT Imaging

Routine cerebral CT images (sequential cCT native, 4.5 mm slice thickness, and supra- and infratentorial; mAs = 50; kV = 120) were acquired on a 16-row multislice CT scanner (Somatom 16; Siemens Medical Systems, Erlangen, Germany). To calculate lesion size, images were analyzed with OSIRIX 5.6. Regions of interest were defined manually, and the lesion volume was calculated semiautomatically.

#### 2.1.4. Blood Sampling

Blood samples were obtained immediately upon admission and then between 6:00 a.m. and 8:00 a.m. on days 1, 3, 5, and 7. Investigators were not blinded for control and stroke patient samples, but they were unaware of stroke severity.

#### 2.1.5. Phenotyping of Human Tregs

Aliquots of 200 *μ*L of whole blood, anticoagulated with ethylenediaminetetraacetic acid (EDTA), were incubated with appropriate combinations of fluorescence-conjugated monoclonal antibodies to stain surface molecules. After lysing of erythrocytes (Buffer A; Human FoxP3 Buffer Set, BD Biosciences, Heidelberg, Germany), cells were washed twice and prepared for intracellular staining without further stimulation. After 30 min incubation with anti-FoxP3-antibodies coupled with Alexa Fluor 647 (BioLegend, San Diego, CA, USA) and an additional washing step, cells were measured on a BD Canto II or BD LSR II flow cytometer (BD Biosciences, San Jose, CA, USA).

The monoclonal antibodies used to determine expression of cell surface molecules were CD25-PE-Cy7, CD49d-FITC, and CD4-V500 (BD Biosciences, Heidelberg, Germany) and CD45RA-PerCP-Cy5.5 and CD39-PE (BioLegend, San Diego, CA, USA). Isotype control antibodies coupled to PE, PerCP-Cy5.5, and Alexa Fluor 647 were from BioLegend and those coupled to FITC, PE-Cy7, and V500 were from BD Biosciences.

For each stroke patient or control sample six appropriate fluorescence minus one (FMO) controls were prepared to identify positive events by flow cytometry. To account for any nonspecific binding of the epitope-specific antibody the appropriate isotype control was added to each FMO control.

Tregs were quantified as CD4^+^CD49d^−^FoxP3^+^ cells. Human proinflammatory effector cells can be transiently FoxP3^+^ but bear CD49d. Therefore, CD49^+^ cells were excluded from the FoxP3^+^ population [27]. Within this population of Tregs, expression of CD45RA was used to identify naive Tregs, while CD39 surface expression was used to detect activated Tregs. There were no CD39^+^CD45RA^+^ cells; however, there was a consistent population of CD39^+^CD45RA^−^ Tregs. Flow cytometry results were evaluated by FlowJo software 7.6.5 (Tree Star Inc., Ashland, OR, USA).

#### 2.1.6. Isolation of Human Tregs

Tregs were isolated with the CD4^+^CD25^+high^CD127^−/dim^ Regulatory T Cell Isolation Kit II (human) (Miltenyi Biotec GmbH, Bergisch Gladbach, Germany) according to the manufacturer's instructions. In brief, peripheral blood mononuclear cells (PBMCs) were isolated with a Ficoll gradient (Biochrom AG, Berlin, Germany). The CD4^+^CD25^+^CD127^−^ T Cell Biotin-Antibody Cocktail II was used to negatively enrich for CD4^+^CD127^−^ cells followed by a positive selection of CD25^+^ cells.

To assess the purity of these CD4^+^CD25^+high^CD127^−/dim^ cells, samples and PBMCs were tested by flow cytometry. Hence, cells were incubated with the appropriate amount of extracellular antibody and prepared for intracellular staining with FoxP3-Alexa Fluor 647 (BioLegend) using the Human FoxP3 Buffer Set (BD Biosciences). For extracellular staining, CD25-PE-Cy7, CD4-FITC (both BD Biosciences), and CD127-Pacific Blue (BioLegend) were used. In all experiments, the purity of CD4^+^CD25^+high^CD127^−/dim^FoxP3^+^ cells was ≥95%.

#### 2.1.7. Suppression Assay

Once the Tregs were isolated, they were incubated with 100 000/well PBMCs in different ratios (Treg : PBMC: 1 : 1, 1 : 2, and 1 : 4) in a flat-bottomed plate. In accordance with the instructions of the BD FastImmune Human Regulatory T Cell Function Kit (BD Biosciences), cells were activated with an appropriate amount of CD3/CD28 beads (Dynabeads Human T Activator CD3/CD28; Invitrogen, Carlsbad, CA, USA) and incubated with CD154-APC antibodies. After 7 h of incubation at 37°C and 5–7% CO_2_, cells were additionally stained with CD69-PE-Cy7, CD4-FITC, CD25-PE, and CD3-PerCP-Cy5.5 (BD FastImmune Human Regulatory T Cell Function Kit; BD Biosciences). Hence, CD25 served to discriminate Tregs from Teffs in this assay, since the molecule is constitutively expressed on the former but not yet induced within the 7 h activation period in the latter.

Samples were measured by flow cytometry on a BD LSR II (BD Biosciences) in accordance with the company's advice. T cell activation was evaluated on two T effector cell (Teff) populations, on CD25^−^CD3^+^CD4^+^ T helper cells and on CD25^−^CD3^+^CD4^−^ cells, which are mainly CD8^+^ T cells and are therefore referred to as cytotoxic T cells within this paper. The suppressive capacity of the added CD4^+^CD25^+high^CD127^−/dim^ Tregs was determined as inhibition of CD69 and CD154 expression on the Teff populations.

### 2.2. Animal Studies

#### 2.2.1. Animals

All animal experiments were approved by the local government authorities (Landesamt für Landwirtschaft, Lebensmittelsicherheit und Fischerei (LALLF), Mecklenburg-Vorpommern). DEREG (Depletion of regulatory T cells) mice were bred in our animal facility (Zentrale Service- und Forschungseinrichtung für Versuchstiere (ZSFV), Greifswald). DEREG mice carry a DTR-eGFP (DTR: diphtheria toxin receptor, GFP: green fluorescent protein) transgene under the control of an additional FoxP3 promoter allowing Treg depletion by low dose diphtheria toxin injection [25]. Here, we only took advantage of the eGFP expression of Treg for the identification of the subset and did not deplete Treg cells.

#### 2.2.2. Ischemia Model

Male undepleted DEREG mice of different ages (8–46 weeks) underwent left MCAO using the filament model. Anesthesia was induced at 2.5% isoflurane with 70% N_2_O/30%O_2_ and maintained at 2% isoflurane with 70% N_2_O/30%O_2_ during surgery. Body temperature was measured with a rectal probe and maintained using a feedback-controlled heating pad for a body temperature of 37°C ± 0.5°C. Briefly, the common carotid artery and the external carotid artery were dissected and ligated. A silicon-coated filament (Doccol Corporation, MA, USA) was introduced into the common carotid artery and advanced into the internal carotid artery until the origin of the middle cerebral artery. The surgery time for ischemia induction did not exceed 15 minutes. The filament was withdrawn after 45 min occlusion time. Body weight and body temperature were measured according to protocol.

#### 2.2.3. Mouse MRI

For 7T-animal MRI (ClinScan, Bruker Biospin, Ettlingen, Germany) mice were anesthetized with 1-2% isoflurane and 1 L/min oxygen. During brain scans respiration was monitored and animals were kept warm using an external water bath. For brain scans at day 1 after MCAO a 3D-T2 weighted imaging (mouse brain coil, TR = 2000 ms, TE = 37 ms, FoV 19 × 25 mm, thickness 0.45 mm) and additional diffusion weighted imaging for visualization of the acute infarct were performed. For evaluation of T2 lesion volume of the brain MRI data were analyzed by two independent investigators with respect to lesion location and size. Regions of interest were selected manually and the volume was calculated semiautomatically using OsiriX software.

#### 2.2.4. Flow Cytometry of CD39 Expression on Murine Treg

CD39 expression was determined on FoxP3 expressing Treg of naive (8–48 weeks old) and stroked undepleted DEREG mice. Transient MCAO was described before. Similar to naive mice, on d3 following stroke blood was withdrawn directly from the heart and anticoagulated with EDTA. Spleens were then collected after transcardial perfusion with ice-cold 0.9% saline in deeply anesthetized mice. After homogenization and hypotonic lysis of red blood cells, the single cell suspension was used for flow cytometric analysis of CD39 expression on CD4^+^ FoxP3^+^ Treg. Unwanted Fc receptor staining was blocked by initial incubation of cell suspensions with TruStainFcX13 (anti-mouse CD16/32, BioLegend, San Diego, CA, USA). Tregs were identified by anti-CD4-Brilliant Violet 605 antibody (BioLegend, San Diego, CA, USA) and the transgenic expression of eGFP under control of the FoxP3 promotor. CD39 surface expression was determined on CD4^+^FoxP3^+^ lymphocytes by staining of CD39 with an anti-CD39-PE antibody (BioLegend) and its isotype control IgG2a PE (BioLegend, San Diego, CA, USA). Flow cytometry was performed on a Becton Dickinson LSRII. Data were analyzed using FlowJo (Tree Star Inc., OR, USA).

### 2.3. Statistical Analysis

All datasets were tested for deviations from Gaussian distribution with the Kolmogorov-Smirnov test. Data that passed the test were analyzed by repeated-measures analysis of variance (ANOVA), with Bonferroni's multiple-comparison test as a posttest. Since some of the data in each* in vitro* experiment failed the normality test, we used nonparametric testing throughout. The Kruskal-Wallis test, with Dunn's multiple-comparison test as a posttest, was used as appropriate. Posttests were only performed if the initial testing revealed significant differences between the groups. Correlations were determined by Pearson correlation analysis. Tests were performed with 95% confidence intervals (two tailed). All analyses were carried out with the software GraphPad-PRISM 5.0 (GraphPad Software Inc., San Diego, CA, USA). A *p* value of <0.05 was regarded as significant.

## 3. Results

### 3.1. CD39 Expression on Tregs Increases with Age

**Figure 1:**
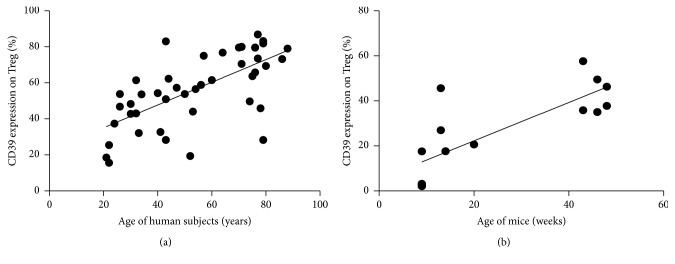
CD39 expression on Treg correlates positively with age in man and mice. (a) Human, peripheral blood: the percentage of CD39 expression was determined on CD4^+^CD25^+^CD49d^−^FoxP3^+^ Treg and correlated to age for a total of 45 healthy controls (age from 21 to 88 years). Pearson *r* = 0.6612, *p* < 0.0001, and* R* squared = 0.4372. (b) Mice, blood: the percentage of CD39 expression was determined on CD4^+^CD25^+^CD49d^−^FoxP3^+^ Treg and correlated to age for a total of 15 naive animals (age from 8 to 48 weeks). Pearson *r* = 0.7895, *p* < 0.0005, and* R* squared = 0.6233.

Since it is not known how age affects the CD39 subset of Tregs we determined the age dependent expression of CD39 on CD49^−^FoxP3^+^ Tregs. In our population the percentage of Tregs of CD4^+^ T cells did not change significantly, while the percentage of CD45RA expressing naive Tregs declined with age (*r* = −0.8422; *p* < 0.0001) (Supplemental Figure S1) (see Supplementary Material available online at http://dx.doi.org/10.1155/2016/2974605). CD39 expression increased with age (*r* = 0.6612; *p* < 0.0001) (Figure 1(a)).

### 3.2. Tregs Are Reduced in the Peripheral Blood of Stroke Patients

**Figure 2:**
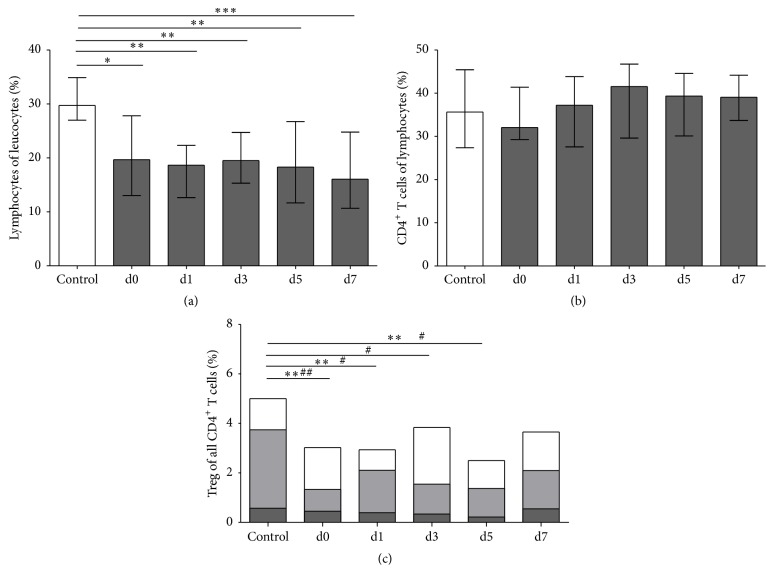
T helper cells and Tregs and their subpopulations. Cytometric analysis of nonstroke controls (white bars) and stroke patients (dark grey bars) was performed for (a) lymphocytes (*n*_control,d0,1,3,5,7_ = 15, 35, 35, 35, 32, 30, resp.), shown as the percentage of leukocytes. ^*∗*^ *p* < 0.05, ^*∗∗*^ *p* < 0.01, and ^*∗∗∗*^ *p* < 0.001. (b) CD4^+^ T helper cells (*n*_control,d0,1,3,5,7_ = 15, 35, 35, 35, 32, 29, resp.), shown as the percentage of lymphocytes. (c) Total Tregs (*n*_control,d0,1,3,5,7_ = 15, 35, 35, 34, 32, 27, resp.) combined with their subpopulations. Naive Tregs (*n*_control,d0,1,3,5,7_ = 15, 34, 35, 33, 31, 27, resp.) are shown by dark grey bars and activated Tregs (*n*_control,d0,1,3,5,7_ = 14, 35, 34, 33, 32, 27, resp.) by light grey bars. Patient samples were obtained on admission (d0), the next morning (d1), and on days 3, 5, and 7 (d3, d5, and d7, resp.). ^*∗∗*^ *p* < 0.01 for Tregs in stroke patients versus controls; ^#^ *p* < 0.05 and ^##^ *p* < 0.01 for activated Tregs in patients versus controls. Medians and interquartile ranges (a, b) are provided.

Flow cytometric analysis confirmed the well-described loss of lymphocytes in the peripheral blood of stroke patients, which was highly significant on all days (*p* = 0.0035). The proportion of CD4^+^ T cells to total lymphocytes was not significantly altered in this stroke patient cohort, indicating that CD4^+^ T cells are lost in a similar quantity from peripheral blood as CD4^−^ lymphocytes (Figure 2). Total Tregs, which accounted for 5.0% (median) (range 1.3–10.2%) of CD4^+^ T cells in healthy controls, were reduced in stroke patients to 2.8% (median) (range 0.03–8.1%) on admission and remained below control values until day 7 (*p* = 0.0095). This reduction was due to a loss of CD39^+^ activated Tregs (*p* = 0.0335), which reached a minimum of 1.2% (median) (range 0.2–7.1%) of all CD4^+^ T cells on day 5. Naive Tregs, which express CD45RA, remained largely unchanged (Figure 2(c)). In controls, 92.1% median (range 71.0–98.3%) of Tregs expressed the activation marker CD25. In stroke patients, CD25 expression was not significantly altered (*p* = 0.0600) (Supplemental Figure S2A).

We also assessed the percentage of CD4^+^CD25^+^ cells in stroke patients, as this has been used previously as a marker for Tregs; however, activated T cells also upregulate CD25 on their cell surface. Stroke patients had 30.4% (median) (range 4.2–71.6%) CD4^+^CD25^+^ cells on the day of admission, which did not differ significantly from control values 41.2% (median) (range 5.1–73.7%) (*p* = 0.7886) (Supplemental Figure S2B).

No differences were seen when we compared the Treg populations between sexes. There was no robust correlation between percentage of Tregs and their subpopulations with respect to the neurological deficit or stroke size.

### 3.3. Age Dependent Regulation of CD39^+^ Treg in Mice

**Figure 3:**
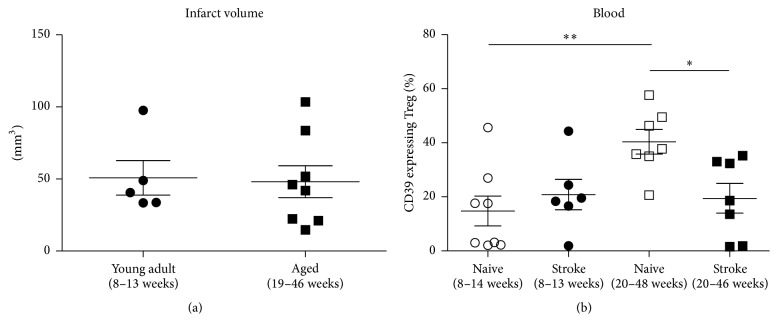
Age dependency of infarct volume and CD39 expression on Treg after experimental stroke in mice. (a) The infarct volume as assessed by MRI in young adult and aged mice was compared on day 1 after transient middle cerebral artery occlusion. (b) The CD39 expression on CD4^+^CD25^+^CD49d^−^FoxP3^+^ Treg in blood was compared between naive and stroked young adult and naive and stroked aged mice on day 3 after transient middle cerebral artery occlusion. ^*∗*^ *p* < 0.05, ^*∗∗*^ *p* < 0.01. Means and SEM are provided.

Using nondepleted DEREG animals the GFP expression of FoxP3^+^ Treg was used to quantify peripheral blood Treg. In aged mice the proportion of CD39 expressing Treg was increased compared to young adult mice (Figure 1(b)). Induction of cerebral ischemia by transient filament MCAO resulted in similar infarct sizes in young and aged mice (Figure 3(a)). Nevertheless CD39 expression on Treg in SIIA differed between young and aged animals. While there was no effect on CD39 expressing Treg in young mice the age related increase in CD39 expression in peripheral blood was reversed by MCAO in aged mice (Figure 3(b)).

### 3.4. Impaired Treg Function in Stroke Patients

Treg function was quantified by inhibition of the activation-induced upregulation of CD69 and CD154 on the surface of Teffs. We therefore compared this activation marker expression on T helper cells and cytotoxic T cells derived from stroke patients and controls. Directly* ex vivo* T helper cells from stroke patients expressed more CD69 on their surface (*p* = 0.0243) compared to controls, while CD154 expression did not differ between the groups (*p* = 0.4140) (data not shown). There was no difference in the expression of CD69 or CD154 on cytotoxic T cells from control versus stroke patients (CD69, *p* = 0.2238, and CD154, *p* = 0.1449). Upon CD3/CD28 activation* in vitro*, no differences in CD69 or CD154 expression on the cell surface of either CD4^+^ T cells or CD8^+^ T cells were seen between stroke patients and controls (data not shown).

**Figure 4:**
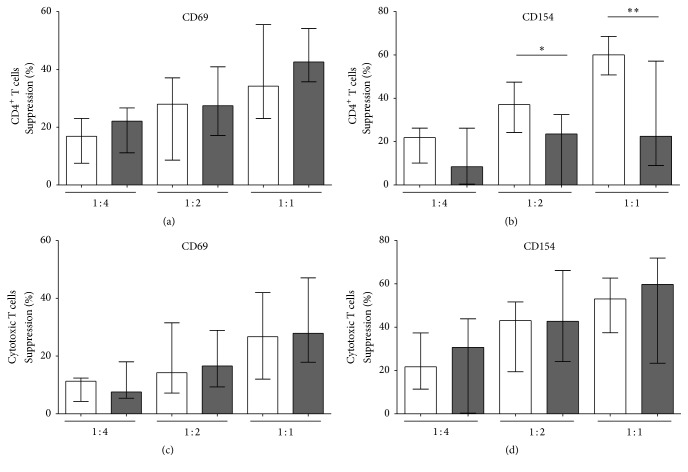
Suppressive activity of Tregs. Treg function was evaluated by measuring the CD4^+^CD25^+^CD127^dim/−^ Treg-mediated inhibition of CD69 (a, c) and CD154 (b, d) induction on T effector cells (Teff) after anti-CD3/anti-CD28 stimulation. Suppression is shown for T helper cells (a, b) and cytotoxic T cells (c, d) in control subjects (white bars) versus stroke patients (dark grey bars) on day 3 with different Treg : PBMC concentrations (Treg : PBMC = 1 : 4; 1 : 2; and 1 : 1). *n*_control,stroke  on  day  3_ = 11 and 10, respectively. ^*∗*^ *p* < 0.05 and ^*∗∗*^ *p* < 0.01 for stroke patients versus controls. Medians and interquartile ranges are provided.

Treg-mediated inhibition of T helper cell activation was impaired in stroke patients. At a ratio of 1 : 2 and 1 : 1 Tregs : PBMCs, the inhibition of CD154 expression on CD4^+^ effector cells was reduced in stroke patients compared to healthy controls (*p* < 0.0001) (Figure 4(b)). Inhibition of the early-activation marker CD69 on T helper cells remained unaltered (Figure 4(a)). The effect of Tregs on cytotoxic T cells was not altered in stroke patients (Figures 4(c) and 4(d)).

**Figure 5:**
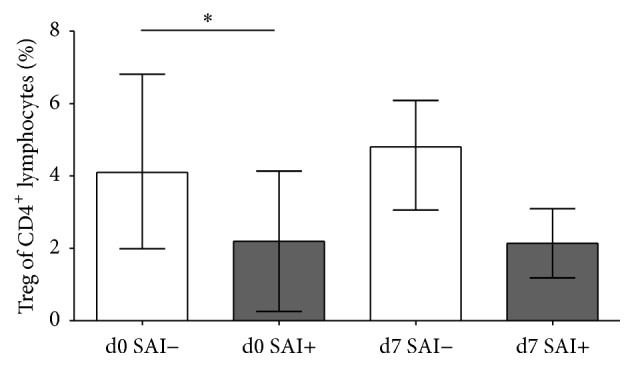
Comparison of SAI− and SAI+ patients. On the day of admission (d0) and day 7 after stroke (d7) the percentage of Treg of CD4^+^ T helper cells was compared between patients without poststroke infections SAI− (white bars;* n*_d0,d7_ = 14, 12, resp.) and patients with poststroke infections SAI+ (grey bars;* n*_d0,d7_ = 7, 7, resp.). ^*∗*^ *p* < 0.05. Means and SD are provided.

Since Yan et al. detected no changes in Treg in a cohort of stroke patients that remained free of infection throughout the hospital stay, we reanalyzed our data according to infection status [19, 26]. As shown in Figure 5 the effect of stroke on total Treg and subsets was more pronounced in those patients that went on to develop SAI during their hospitalization.

## 4. Discussion

In this study, we found decreased numbers and impaired function of Tregs in the peripheral blood of stroke patients. The decrease in Tregs following stroke was detectable upon admission and remained statistically significant through day 5. This reduction in Tregs was not evenly distributed among all FoxP3^+^ Treg subsets but was most pronounced in the active CD39^+^ Treg population.

The reduced efficacy of Treg-mediated Teff suppression in stroke patients is in agreement with reports by Yan et al., who found a similar suppression using proliferation as a read-out [19, 26]. However, a study by Hug et al. was unable to detect altered Treg function in stroke patients [5]. In this previous study, no dose-response curve was obtained and the suppression measurable using Tregs and Teffs of control individuals was only 10%. Therefore, the conditions chosen by Hug et al. for the assay may not have been sufficiently sensitive to detect impaired Treg function. We applied an assay that is based on activation marker expression on T responder cells rather than proliferation. The validity of inhibition of CD154 expression on Teff as a marker for Treg suppressive activity has been demonstrated [27]. This approach enabled us to distinguish between the effect of Treg on CD4^+^ and CD4^−^ Teff.

Since all three studies used autologous Teff cells as responder cells, an enhanced resistance of Teff to Treg-mediated inhibition cannot be excluded. We observed a predominant loss of CD39 expressing Tregs, which represents the functionally active Treg subset, with a concomitant shift in Treg composition toward the naive subset. It is therefore plausible that Treg function rather than Teff susceptibility is impaired in stroke patients.

Our data corroborate recent observations by Li et al., who detected a reduced percentage and suppressive activity of Tregs in stroke patients, and extend their findings by demonstrating that functionally active CD39^+^ Tregs are predominantly reduced following stroke [28]. Furthermore, to our knowledge, this is the first study to address the inhibition of activation of cytotoxic Teffs in stroke patients. In contrast to the impaired inhibition of T helper cells, reduction of cytotoxic Teff activation was not altered in stroke patients.

Our quantitative findings, however, are in contrast to two reports by Yan et al., who detected an increase in FoxP3^+^ Tregs following stroke [19, 26]. These differences may be related to the stroke population recruited; in our study all patients had MCA ischemia and an NIHSS ≥6, whereas Yan et al. recruited all ischemic stroke patients, including those who were less severely affected. More importantly, Yan et al. excluded patients with “acute infections after stroke.” Since SAIs are more likely to occur in patients with stroke-induced immunosuppression [2], this approach is likely to exclude patients with the most severe stroke-induced immune alterations. In our study, we also excluded patients with signs of infection on admission; however, patients with subsequent SAIs were not excluded. To evaluate whether this could account for the apparent contradiction, we performed a subanalysis comparing patients with and without subsequent infections. While failing to reach statistical significance, Tregs appeared to be more strongly reduced in patients with SAI compared to those without SAI. This supports our hypothesis that the differences in patient populations may account for the seemingly contradictory findings between our findings and the data reported by Yan et al.

The major limitation of the study is the fact that our analysis is restricted to the peripheral blood as other immune compartments are not readily accessible in patients. Therefore we cannot exclude that the active Tregs have migrated into the tissues. Our animal data indicate that aged mice could provide a suitable model to determine the fate of Treg subsets following stroke. Immunosenescence has been shown to alter the clinical course of diseases and also affect Treg subsets [29–31]. We extend this knowledge by demonstrating that the active Treg population circulating in the peripheral blood increases with age. The role of Treg in regulating SIIA remains disputed and differs between the experimental models [14–18]. Our data suggest that Tregs are regulated differently during immunosenescence, an aspect rarely reflected in current experimental stroke models.

## 5. Conclusion

Our data demonstrate that CD4^+^CD49^−^Foxp3^+^ Tregs are reduced in the peripheral blood of stroke patients. Among those the active CD39^+^ Treg subset is the most affected, a finding mirrored in functional studies demonstrating impaired suppressive activity of stroke patient derived Treg* in vitro*. Whether these alterations contribute to secondary immune-mediated brain damage or whether reduced Treg function is beneficial in stroke remains to be investigated. The observation that the proportion of Treg expressing CD39 increases with age and is differentially regulated in young adult and aged mice highlights the importance to consider immunosenescence in the design of experimental stroke models.

## Supplementary Material

The percentage of CD45RA expressing naive Tregs declined with age in humans (*r*=-0.8422; *p*<0.0001) (Fig. S1).
In stroke patients, CD25 expression was not significantly altered (*p*=0.0600) (Fig. S2A).
We also assessed the percentage of CD4+CD25+ cells in stroke patients, as this has been used previously as a marker for Tregs; however, activated T cells also upregulate CD25 on their cell surface. Stroke patients had 30.4% (median) (range 4.2–71.6%) CD4+CD25+ cells on the day of admission, which did not differ significantly from control values 41.2% (median); (range 5.1–73.7%) (*p*=0.7886) (Fig. S2B).

## Figures and Tables

**(a):** 

	Total number	Age^†^	NIHSS^‡^	Lesion volume (mm^3^)^§^	Localization of infarction	Thrombolysis/ thrombectomy	Male	Female
Control subjects	26	69,5 (51–88)	NA	NA	NA	NA	12	14

Stroke patients	48	77 (55–93)	13 (23–6)	71,5 (4,99–1022)	45 MCA 3 MCA + anterior	17	16	32

**(b):** 

	Total number	Age^†^	NIHSS^‡^	Lesion volume (mm^3^)^§^	Localization of infarction	Thrombolysis/ thrombectomy	Male	Female
Noninfected cohort (SAI−)	15	77 (62–93)	8 (6–23)	44,05 (4,99–1022)	15 MCA 1 MCA + anterior	4	5	10

Infected cohort (SAI+)	7	74 (55–87)	17,5 (13–19)	174,24 (18,52–318,27)	7 MCA	2	4	3

^†^Mean (range); ^‡^median (range); ^§^median (range); NA: not applicable; MCA: middle cerebral artery; SAI: stroke associated infection.
