# Supplementary material for: Reduced Numbers and Impaired Function of Regulatory T Cells in Peripheral Blood of Ischemic Stroke Patients
Source: Mediators Inflamm. 2016 Mar 17;2016:2974605. doi: 10.1155/2016/2974605 (PMC4814689; doi:10.1155/2016/2974605)
Supplement: Supplementary file 1 — The percentage of CD45RA expressing naive Tregs declined with age in humans (r=-0.8422; p<0.0001) (Fig. S1). In stroke patients, CD25 expression was not significantly altered (p=0.0600) (Fig. S2A). We also assessed the percentage of CD4+CD25+ cells in stroke patients, as this has been used previously as a marker for Tregs; however, activated T cells also upregulate CD25 on their cell surface. Stroke patients had 30.4% (median) (range 4.2–71.6%) CD4+CD25+ cells on the day of admission, which did not differ significantly from control values 41.2% (median); (range 5.1–73.7%) (p=0.7886) (Fig. S2B). [file 2974605.f1.pdf]

## Supplement

Fig. S1

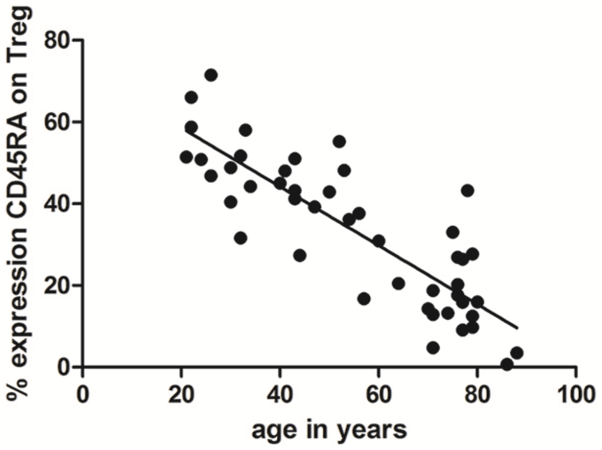


**Suppl. Figure S1.** CD45RA expression on Treg correlates negatively with age in humans.

Human, peripheral blood: The percentage of CD45RA expression was determined on $CD4^{+}CD25^{+}CD49d^{-}FoxP3^{+}$ Treg and correlated negatively to age for a total of 45 healthy controls (age from 21 to 88 years). Pearson r=-0.8422; p<0.0001

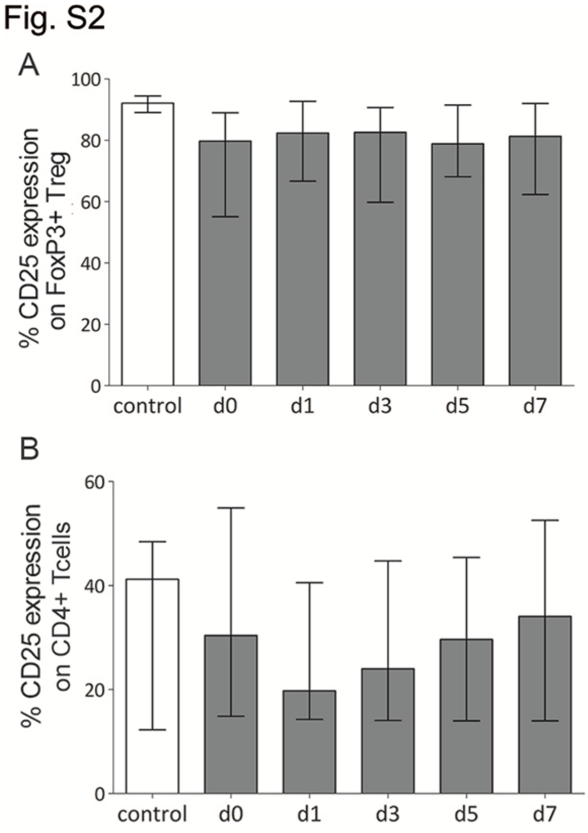


**Suppl. Figure S2.**

A) Expression of CD25 on cell surface of Treg

Cytometric analysis of nonstroke controls (white bars) and stroke patients (dark grey bars) were performed for CD25$^+$ expressing Treg ($n_{\text{control, d0, 1, 3, 5, 7}}$ = 15, 35, 35, 34, 32, 22), shown as the percentage of FoxP3$^+$cells. Medians and interquartile ranges are provided.

B) Analysis of CD4$^+$CD25$^+$ cells

Analysis of nonstroke controls (white bars) and stroke patients (dark grey bars) were performed for CD25$^+$ expressing CD4$^+$ cells ($n_{\text{control, d0, 1, 3, 5, 7}}$ = 14, 35, 34, 33, 32, 25), shown as the percentage of CD4$^+$cells. Medians and interquartile ranges are provided.
